# Effects of the lockdown period on the mental health of elite athletes during the COVID-19 pandemic: a narrative review

**DOI:** 10.1007/s11332-022-00964-7

**Published:** 2022-06-08

**Authors:** Vittoria Carnevale Pellino, Nicola Lovecchio, Mariangela V. Puci, Luca Marin, Alessandro Gatti, Agnese Pirazzi, Francesca Negri, Ottavia E. Ferraro, Matteo Vandoni

**Affiliations:** 1 Laboratory of Adapted Motor Activity (LAMA)- Department of Public Health, Experimental Medicine and Forensic Science, University of Pavia, Pavia, Italy; 2 Department of Industrial Engineering, University of Tor Vergata Rome, Rome, Italy; 3 Department of Human and Social Science, University of Bergamo, Bergamo, Italy; 4 Unit of Biostatistics and Clinical Epidemiology, Department of Public Health, Experimental Medicine and Forensic Science, University of Pavia, Pavia, Italy; 5 Laboratory for Rehabilitation Medicine and Sport (LARMS), 00133 Rome, Italy; 6 Department of Research, ASOMI College of Sciences, Marsa, 2080 Malta

**Keywords:** COVID-19, Lockdown, Elite athletes, Psychological distress, Mental health

## Abstract

**Purpose:**

This review aimed to assess the effects of COVID-19 pandemic lockdown on mental health to elite athletes. The emotional background influenced their sport career and was examined by questionnaires.

**Methods:**

We included original studies that investigated psychological outcomes in elite athletes during COVID-19 lockdown. Sixteen original studies (*n* = 4475 participants) were analyzed.

**Results:**

The findings showed that COVID-19 has an impact on elite athletes’ mental health and was linked with stress, anxiety and psychological distress. The magnitude of the impact was associated with athletes’ mood state profile, personality and resilience capacity.

**Conclusion:**

The lockdown period impacted also elite athletes’ mental health and training routines with augmented anxiety but with fewer consequences than the general population thanks to adequate emotion regulation and coping strategies.

## Introduction

The outbreak of SARS-Cov-2 (COVID-19) and the ongoing pandemic caused a public health concern all over the world with health, social, and economic negative consequences [1, 2]. To limit the spread of the virus, governments were forced to impose lockdown measures with the “stay at home” imperative. Everyday life changed all over the world: social-distancing, mask-wearing, limited travel, leisure activity, and non-essential activities stopped [3]. These restrictions affected the entire population promoting sedentary behavior and inactive lifestyle [4, 5], which led to acute and long-term physical [6] and mental disorders, such as acute stress disorder, exhaustion, irritability, insomnia, poor concentration, indecisiveness, fear, and anxiety [7–9]. For these reasons, several researchers and studies implemented and provided specific recommendations for general population health [2, 10] and fitness [11] to better cope COVID-19 period. The sports contest was not excluded from the protective measures against pandemic and, at every level, it was affected by an extraordinary period with the closure of training facilities, and the interdiction of training both for amateurs and elite athletes until all sports’ competitions postponement (e.g. Olympic Games) or cancelation [7, 8]. Even if the priority remains the limitation of contagion, the imposed restrictions did not allow athletes to follow their training and competitive routines, because they were forced to train at home, on their own, and often with no trainers supervision. For this reason, specific suggestions have also been provided for elite and professional athletes [12] to maintain health, optimal body composition, specific routine exercise, physical conditioning, to encourage a safe return to training and competitions, and to avoid psychological distress (according to the American Psychology Association dictionary, “a set of painful mental and physical symptoms that are associated with normal fluctuations of mood in most people”) [12–16].

Previous studies showed that a long-term detraining, of at least eight weeks as a similar effect due to COVID-19 forced to stop, leads to a marked decline in maximal oxygen consumption (VO2max), endurance capacity, and muscle strength and power with a reduction of electromyography activity (EMG) that reflects reduced muscle activation [17]. All these declines in athletes’ physical condition have been shown to significantly increase the risk of injuries, fear of return to competition, and psychological distress [18]. Moreover, in this last period, many athletes have reported challenges and issues connected to social isolation, like career disruption or uncertainty of contract status, and ambiguity of the qualification process, which could be additional stressors and could increase psychological distress, affecting the training and the performance of the athletes [19, 20]. Consequently, the awareness of stress and anxiety outcomes became relevant for sports specialists, coaches, and sports psychologists to help athletes to maintain focus, motivation, coping strategies and find, organize, and plan the best strategies for return to competition without fears.

Before the COVID-19 pandemic, an elite athlete encountered a lot stressors during the career [21], the COVID-19 restrictions seems to have amplified all the stressors with negative consequences on the mental health of athletes. Unfortunately, the present literature does not seem to clarify the possible causes and effects of COVID-19 restrictions on athletes. So, the present narrative review aims to describe how the COVID-19 pandemic lockdown influenced the mental health of elite athletes. Specifically, the primary objective of this review is to identifies the common psychological distress and stress responses on elite athletes during the COVID-19 pandemic. Second, our research aims to identify factors, either positive or negative, related to psychological distress in elite athletes during the COVID-19 pandemic.

## Methods

### Search strategy

The study was conducted up to 24th November 2021 through computerized research in the databases PubMed, Scopus, SportDiscus and Web-of-Science for papers published in English in peer-reviewed journals providing information related to mental health of athletes during COVID-19 lockdown.

The following search terms were used: (coronavirus OR COVID-19 OR lockdown OR isolation) AND (sport competition OR sport participation OR training) AND (elite athletes OR athletes OR collegiate athletes) AND (mental health OR psychological distress) AND (COVID-19 OR elite athlete OR mental health). Finally, the reference lists of the studies were manually checked to identify potentially eligible studies not found by the electronic searches. Two reviewers independently: (a) screened the title and abstract of each reference to determine potentially relevant studies, and copies of the screened documents were obtained; (b) subsequently, reviewed them in detail to identify articles that met the inclusion criteria. Third reviewer solved discrepancies between reviewers in the studies selection.

### Study selection criteria

**Fig. 1:**
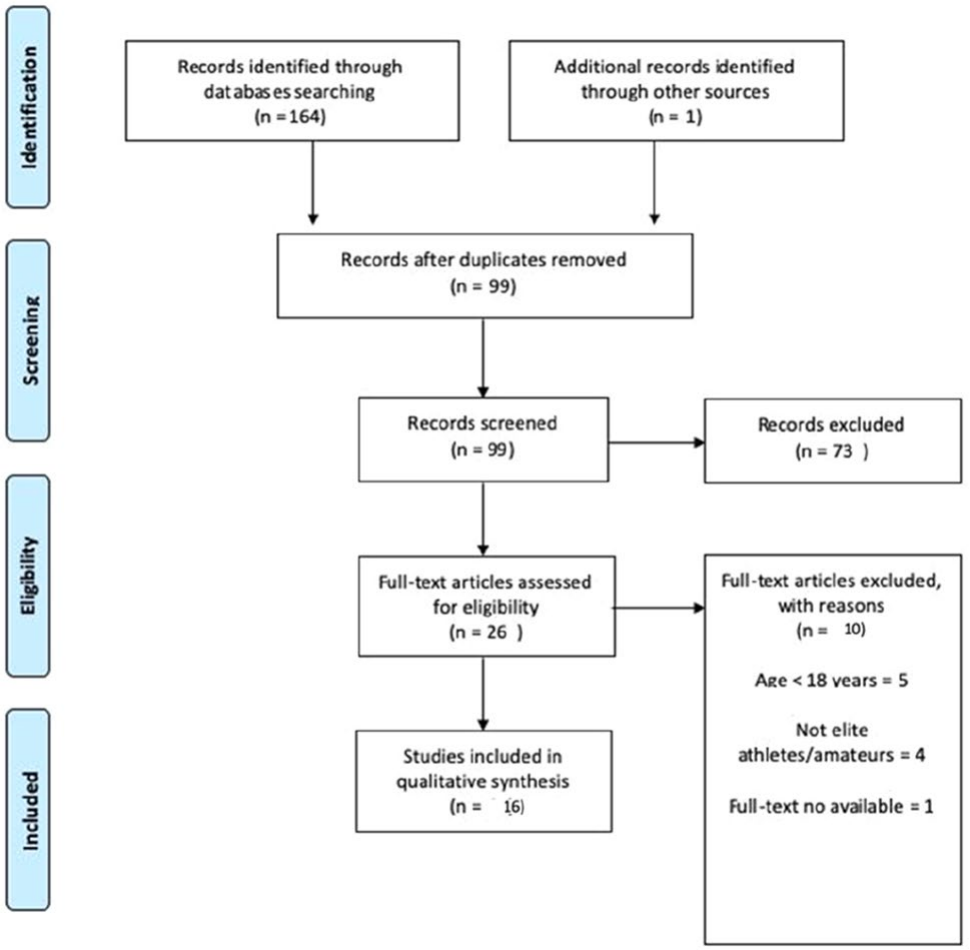
Study selection flow-chart

Research articles were included if they fulfilled the following criteria: (1) studies with full-text available and had to investigated mental health in athletes with elite or professional or international/national status older than 18 years old pre and during COVID-19 pandemic; the elite status of the athletes was given to the athletes with at least 5-day per week training and participation into national and international sport-specific competitions; (2) studies had to specify the duration of lockdown period; (3) studies had to assess psychobiological factors through valid and reliable tools/questionnaires or showed full questions and scored. Finally, we excluded narrative reviews, abstracts, editorial or commentaries, letters to the editors and case reports and studies that investigated athletes under quarantine or ongoing COVID-19 infection. Flow chart of included studies is shown in Fig. 1. All the main findings was synthetized through a narrative approach [22].

## Results

### Descriptive and methodological characteristics of the studies

The characteristics of the included studies were summarized in Table 1. After the screening, sixteen studies were included in this review; four were carried out in Italy, three in Spain, one in the United States, one in France, one in Portugal, one in Estonian, one in Sweden, one in Iran, one in Australia, one in Poland and one in South Africa. All studies had cross-sectional designs. Due to COVID-19 restrictions, all studies’ data were collected online through specific survey platforms (*n* = 11), mails or WhatsApp (*n* = 2), professional networks (*n* = 3). Data collection was conducted between 9th March 2020 and 31st August 2020 during the initial phases of COVID-19 lockdown. All the studies recruited athletes in specific clusters (national and international level, Olympic qualifiers, professionals). Of sixteen studies, eight focused explicitly only on elite athletes, while seven studies were conducted both in amateur and elite athletes and then the subjects' outcomes were analyzed separately. The last one includes athlete affiliate to national federation, coaches and managers. Of sixteen studies, thirteen focused on participants aged 18 or older while three studies reported data also for < 18 years old; this data were excluded from the analysis. In total, 4475 elite athletes were surveyed, where the sample size of each study varied from 57 to 692 participants. All the studies reported gender distribution of the whole sample but only four studies specified gender distribution for elite athletes. In terms of the measured outcomes, fourteen studies used validated questionnaires while two studies used tools developed by authors. Of the sixteen studies that employed standardized measures nine reported reliability coefficients (Cronbach’s alpha) for their samples, which was satisfactory from *α* = 0.66 to *α* = 0.93.

**Table 1:** Characteristics of the included studies in the review

Authors	Country	Period and methods of data collection	Sample and sport participation	Outcomes	Analytical approach	Mental health outcomes and correlates
Costa S. et al. [37]	Italy	April–May 2020 Contacted through professional software provided by National federation	1125 athletes (610 w) > 18yrs; 572 elite athletes Basketball, beach volleyball, boxing, climbing, cycling, dance sport, fencing, field hockey, futsal, golf, horse riding, martial arts, gymnastic, rowing, rugby, running, shooting gallery, skating, soccer, swimming, tennis, volleyball, weightlifting	Socio-demographic questionnaire, AIMS, Italian version CERQ	Descriptives; CFA; MANOVA 2 × 2x2 Matrix	**↑** AIMS, in particular social identity and negative affectivity **(**Pearson correlation 0.73–0.83) **↑** “putting thing into perspectives” and “rumination” for women. **↑** “planning” and “blame other” for Men. **↑** tendence to “ruminate” and “catastrophize” for Who are higher Athletic Identity
Ruffault A. et al. [25]	France	April–May 2020. Conducted through specific software	722 athletes (358 women) > 18yrs 128 elite athletes Badminton, basketball, boxing, fencing, gymnastic, hockey, ice-skating, judo, rowing, rugby, shooting, skiing, soccer, swimming, table-tennis, tennis, track and field, triathlon	Socio-demographic questionnaire; TFAI-2 and SMS-2 were correlated to return to sport	Descriptives; CFA; CFI; Wilcoxon test for categorical variables two groups; Kruskall–Wallis for categorical variables more than two groups; Kendall’s Tau correlation	**↑** “public self-focus” **(*p* < 0.05)**, “external regulation of extrinsic motivation” **(*p* < 0.05)**, and “autonomous and controlled motivation” for elite athletes. **↓** “cognitive anxiety” (p < 0,05) and “extrinsic motivation” for older athletes (*p* < 0.01) **↑** “cognitive and physiological anxiety” and lower scores of “perceived control” for females
Clemente-Suarez V. J. et al. [23]	Spain	March–April 2020. Conducted through professional networks	136 Olympic athletes (103 women) > 18 yrs Athletics, basketball, canoe, cycling, football, golf, hockey, judo, swimming, table tennis, taekwondo, triathlon, volleyball, weightlifting, wrestling	Individual perception of COVID-19 crisis provided by authors. Brief version of big five personality inventory, UCLA Loneliness Scale, AAQ-II, STAI	Descriptives; Group differences by U Mann–Whitney test or Kruskall–Wallis. Bivariate correlation using Spearman	**↑** social alarm and alteration of training routines. Who had higher psychological “inflexibility” had greatest negative feelings. **↑** “neuroticism” and “inflexibility” for female athletes. (*r* = 0.13)↑ anxiety but in line with no-pathological population. (*r* = 0.60)
Jagim A. R. et al. [38]	United States	April–June 2020. Conducted through specific software	105 athletes (74 women) > 18 yrs Football, baseball, soccer, track/cross country, volleyball, basketball, lacrosse, gymnastic, golf, rugby	No validated tools. Survey of 36 questions provided by authors (demographic, training and programming, change in training habits due to COVID-19)	Descriptives; Paired T test	67,6% **↓** motivation to train and 65,7% **↓** training satisfaction. VAS at 51.59 19.59. Lack of in person support could reduce QoL and safety
Pillay L. et al. [33]	South Africa	March–April 2020. Conducted through WhatsApp	692 Athletes (225 women) > 18 yrs Athletics, basketball, cycling, running, golf, hockey, karate, netball, rugby soccer, squash, swimming	No validated questionnaire. Survey of 30 questions (return to sport; training, other activities, nutrition and mental state during lockdown)	Descriptives; Chi-square fit test; Bonferroni post hoc	**↑** sedentary behavior, changes in diet and sleep–wake times; 52% felt depressed (W > M); 55% struggled to keep motivated in training; 31% felt unsure to return to sport
Di Cagno A. et al. [36]	Italy	March–May 2020. Conducted through specific survey	1508 athletes, of these 368 were elite adults (> 18 yrs) Basketball, gymnastics, volleyball, soccer	Socio-demographic Questionnaire; IES-R	Descriptives; Kolmogorov–Smirnov test for normal distribution. To test differences was used Kruskall–Wallis test. U Mann–Whitney and the Bonferroni alpha as post hoc	30% being distressed. **↑** “Hyperarousal”. Women achieved higher scores on “self-perceived stress” and “emotional avoidance-response behavior”. (*p* = 0.045) ↑ “personal responsibility” and “perseverance” for individual athletes
Di Fronso S. et al. [28]	Italy	April–May 2020. Conducted through professional networks	1132 athletes (595 women). Of these, 665 were elite. Basketball, golf, rugby, soccer, swimming, tennis	Socio-demographic questionnaire; IPSS-10; PBS-S	Descriptives; CFA; Cohen’s d index; Pearson correlation; MANOVA 2 × 2x2 matrix	**↑** perceived stress and dysfunctional states (*p* < 0.01). Women higher level of both
Mon-Lopez D. et al. [31]	Spain	April 2020. Conducted through WhatsApp	187 athletes (66 women). 77 were elite. Handball	Socio-demographic questionnaire, POMS; WLEIS-S; BRS-II	Descriptives; Kolgomorov–Smirnov and Shapiro–Wilk test for normal distribution; Paired sample t-tests to comparison; effect size using Cohen’s d index; Confidence Interval; ANOVA; Bonferroni post hoc test	**↓** training days, volume and hours. **↑** sleep hours. Use of emotion greatest impact on training conditions; **↓** resilience with higher training intensities; **↑** depression to a higher training volume; tension and anxiety greatest negative impact on sleep quality (*p* < 0,001)
Leguizamo F. et al. [24]	Portugal	April 2020. Conducted by professional networks	310 athletes, (141 women) Athletics, basketball, martial arts, rugby, swimming	Socio-demographic questionnaire; FMPS; STAI-T; DASS-21; POMS; ACSQ-21	Descriptives; Kolgomorov–Smirnov test for normal distribution; difference through Kruskall–Wallis; U Mann–Whitney median difference and Spearman’s Rho for correlation	Negative correlation between emotional state perceived as anxiety, depression, stress and fatigue and the use of coping strategies as cognitive structuring and emotional calming. ↑ stress and vigor profile (p < 0,001)
G. Fiorilli et al. [29]	Italy	cognitive survey was administered during the first phase of the Italian lockdown period	only inclusion criterion: affiliate with a national federation and/or a sports association	Socio-demographic questionnaire IES-R	ANOVA tests	subjective distress (34.4%) ↑ stress for female athletes and coaches than the males (*p* = 0.000)
		(March 12, 2020 to May 3, 2020)	1,668 self-selected volunteer: 800 athletes (28.30 ± 10.93 years old); 558 coaches (36.91 ± 11.93 years old); and 310 sports managers (42.07 ± 13.38 years old)			**↑** hyperarousal for the middle-level athletes than the high-level athletes
Amir Hossien Mehrsafar et al. [26]	Iran	after COVID-19 pandemic (spring 2021)	Ninety male professional football players (training: 11.67 ± 1.62 h/week–1; training history: 10.03 ± 3.28 yr) volunteered to participate in our study (age: 26.33 ± 2.48 yr; body mass index: 23.07 ± 2.06 kg m–2)	CSAI-2R FCS CAS	Kolmogorov–Smirnov normality test	significant positive correlations between COVID-19 anxiety and somatic competitive anxiety (cognitive competitive anxiety, and competition). *p* = 0.01 fear of COVID-19 was positively correlated with COVID-19 anxiety. *p* = 0.01 The analysis also indicated that there was no significant correlation between self-confidence with other psychological and physiological variables p > 0.05
Parm et al. [30]	Estonia	Electronic questionnaire ( May–June 2020)	102 Estonian athletes (males = 44)	EST-Q2	EST-Q2 χ^2^ or Fisher Exact test	Most frequently the athletes had above-cutoff scores of fatigue (*p* = 0.021) and insomnia(0.009), followed by symptoms of depression. **↑** distress symptoms for females than males
Leguizamo et al. [24]	Spain	Online questionnaires during April 2020	310 athletes (141 women and 169 men) from different countries in Europe, Asia, and America, and from diverse sports disciplines	FMPS STAI-T short version DASS-21 POMS ACSQ-1 SSQ	Kolgomorov–Smirnov normality test Kursaal Wallis test, median difference analyses for two independent samples using the Mann–Whitney U-test, and correlational analysis using Spearman’s Rho	The results showed that maladaptive perfectionism was related to all the indicators of athletes’ mental health. However, athletes’ levels of anxiety, stress, and depressive symptoms are relatively low, and the use of coping strategies such as cognitive restructuring and emotional calm was associated with lower levels of negative emotional states (p < 0,05)
A. Håkansson et al. [27]	Sweden	From May 21 to handball players and from May 26 to ice hockey players, and the survey closed on June 10	Athletes in top leagues of soccer, ice hockey and handball in Sweden The survey was sent by e-mail, directly only to players who were union members; 487 soccer players (70% men), 140 handball players (67% men) and 518 ice hockey players (96% men), in total of 17 1145 players (82% men)	PHQ-9 GAD-7 PGSI	Chi-squared analyses (or Fisher’s exact test)	Feeling slightly worse during the pandemic was significantly more common in females than in males (*p* < 0.001). Respondents who endorsed depression criteria or anxiety criteria, respectively, were significantly more likely to report worse psychological mood during the COVID-19 crisis and significantly more likely to report worry about their own future in the sport
E. R. Facer-Childs et al. [63]	Australia	The survey was administered online via Qualtrics (Provo, UT, USA) with data collected between May 1 and June 1, 2020	Were surveyed elite and sub-elite athletes (*n* = 565) across multiple sports	MCTQ SDSI PHQ-4 PSS-4 MEQ19	μMCTQ Kruskal–Wallis rank sum tests Kendall–Theil Sen Siegel nonparametric linear regressions	**↓** training frequency along with later mid-sleep time, higher social jetlag, greater sleep latency, and increased screen time before bed are all independently associated with poorer mental health outcomes. (*p* < 0,001)
Marta Szczypińska et al. [32]	Poland	on-line survey on the platform, in the period of April 7–28, 2020 during the COVID-19 pandemic,	7 Polish potential Olympians aged 18 to 39 (M = 26.61; SD = 5.562), including 29 women (52.7%) and 28 men (49.1%) practicing individual sports such as athletics, rowing, fencing, shooting, sport climbing, badminton, swimming, pentathlon, taekwondo, sailing, wrestling, canoeing, judo, cycling, equestrianism and weightlifting	SOC 29 Brief COPE POMS HSQ	Spearman rank correlation coefficients	The results obtained emphasize the importance of positive reframing as a factor contributing to maintaining a positive mood state. The results confirm the importance of factors included in the salutogenic model (sense of coherence, coping strategies) as predictors of athletes' mood during a pandemic (Rho = 0.566)

↑ = increase; ↓ = reduction; ↔ no changes; AIMS = Athletic identity measurement scale

*CERQ* Cognitive Emotion Regulation Questionnaire; *MANOVA* multivariate analysis of variance; *CFA* confirmatory factor analysis; *TFAI-2* three-factor anxiety inventory 2; *SMS-2* sport motivation scale 2; *STAI* state-trait anxiety inventory; *VAS* visual analogue scale; *QoL* quality of life; *IES * impact of event scale—revised; *POMS*  profile of mood state; *WLEIS-S*  Wong law emotional intelligence scale short form; *BRS-II*   brief resilience scale; *IPSS-10*   Italian 10-item version of the perceived stress scale; *PBS-S*   psychobiosocial states scale; *FMPS*  multidimensional perfectionism scale; *AAQ-II*   acceptance and action questionnaire II; *STAI-T*   state-trait anxiety inventory; *ACSQ-1*  approach to coping in sport questionnaire; *DASS-21*  depression, anxiety and stress scale. *POMS*  profile of mood state; *ACSQ-21*  approach to coping in sport questionnaire-21; *IES-R*  impact of event scale; *CSAI-2R*   competitive state anxiety inventory 2—revised; *FCS*   fear of Covid-19 scale; *CAS*  coronavirus anxiety scale; *EST-Q2*   emotional state questionnaire; *FMPS*  multidimensional perfectionism scale; *STAI-T*  state trait anxiety inventory; *ACSQ-1*  approach to coping in sport questionnaire; *SSQ*  sport sleep questionnaire; *PHQ-9*  patient health questionnaire-9; *GAD-7*  general anxiety disorder-7; *PGSI*   problem gambling severity index; *MCTQ*   ultra-short munich chronotype questionnaire; *SDSI*   single daytime sleepiness item; *PHQ-4*   patient health questionnaire-4; *PSS-4*  perceived stress scale-4; *MEQ19*  morningness–eveningness questionnaire item 19; *SOC 29*  Antonovsky’s sense of coherence questionnaire; *Brief COPE*  brief version of the inventory for measuring coping with stress; *HSQ*   hope for success questionnaire

### Mental health outcomes, coping strategies, resilience and athletes’ motivation

As shown in Table 1, mental health outcomes examined by the studies included depression (*n* = 6), anxiety (*n* = 4), stress and psychological distress (*n* = 5). Depression was investigated through DASS-21 (*n* = 2) with non-pathological conditions found, and a specific questionnaire provided by authors (*n* = 1) reported that most of 50% of athletes felt depressed. Depression was also investigated through PHQ-9 and GA by two studies. Anxiety was investigated by four studies, two connected to lockdown restrictions and two related to return to sport. Clemente-Suarez et al. [23] reported higher level of anxiety in Olympic athletes but in line with non-pathological conditions (through STAI short form); Leguizamo et al. [24] reported greater anxiety (through STAI-T) due to uncertainty of competition calendar. Ruffault et al. [25] reported higher level of cognitive anxiety in particular in public self-focus. Mehrsafar et al. [26] showed significant positive correlations between COVID-19 anxiety and somatic competitive anxiety, cognitive competitive anxiety, and competition response. Hakansson et al. [27] underlined that depression and anxiety were associated with feeling worse during the COVID-19 pandemic and with concern over one’s own sports future while Mehrsafar et al. [26] showed significant positive correlations between COVID-19 anxiety and somatic competitive anxiety and competition. In general, females showed higher levels of cognitive and physiological anxiety and lower scores of perceived controls. Stress and psychological distress were investigated by six studies. Di Fronso et al. [28] reported a high level of perceived stress. In this study [28], females reported higher levels of perceived stress. Furthermore, the higher levels of perceived stress was connected to higher dysfunctional and lower functional states and females reported greater levels of dysfunctional states than males. Different studies [24, 27, 29, 30] measured the common symptoms related to stress and reported an increased level of stress symptoms especially in female athletes. Coping strategies (*n* = 1) and resilience (*n* = 1) were also investigated. Leguizamo et al. [24] reported that athletes often use coping strategies to react to situations. In the lockdown period, even if the stress perception increased, athletes that used coping strategies showed lower consequences. Emotional calming and cognitive structuring were the predominant strategies to cope with lockdown. Mon-Lopez et al. [31] reported that athletes’ resilience decreased with higher training intensity. Resilience became a positive predictor of perceived effort during training. Also, Szczypińska et al. [32] highlighted the importance of using adapted coping strategies to reduce stress and improve the mood state with positive reframing instead self-blaming strategy.

Finally, three studies investigated the motivation to continue training (*n* = 1) or related to return to sport competition (*n* = 2). Pillay et al. [33] reported that 55% of investigated athletes struggled to keep motivated to the training. Jagim et al. reported that 67% of investigated athletes had a reduction of level of training motivation with also lower training satisfaction. Ruffault et al. [25] reported that elite athletes had significant higher scores of external regulations of extrinsic motivation to return to sport.

### Athletes’ profile mood state and personality

To determine possible repercussions on mental health related to specific athletes’ profile and personality, five studies investigated the mood state profile (*n* = 2), athletes’ personality (*n* = 1) and perfectionism (*n* = 1). Leguizamo et al. [24] reported an ideal mood profile investigated, in line with the iceberg profile [34] in which the vigor factor is higher than other factors. These results was also confirmed by Szczypińska et al. [32] and Mon-Lopez et al. [31] Clemente-Suarez et al. [23] showed that a predominant of neuroticism personality in athletes led to a worse perception of confinement with higher impact on performance and training routines. Leguizamo et al. [24] also reported high level of perfectionism in elite athletes, in line with existing literature that was associated with higher performance [35].

### COVID-19 emotional reactions

To cope with unique situations caused by COVID-19 lockdown, six studies investigated specific event impact on athlete’s emotion (*n* = 1), acceptance (*n* = 1) and emotion regulation (*n* = 2). Di Cagno et al. [36] reported increased level of hyperarousal activation. In particular, females showed high scores on self-perceived stress as well as in the emotional avoidance-response behavior. Parm et al. [30] and Clemente-Suarez [23] reported higher scores of psychological inflexibility that lead to greatest negative feelings. Costa et al. [37] investigated individual differences in cognitive regulation and found that elite athletes in general had higher levels of “acceptance”, male showed higher levels of “planning” and “blame others” while females showed higher values of “putting things into perspectives” and “rumination”. Mon-Lopez et al. [31] showed that the athletes’ emotional intelligence impacted on training conditions and capacity to react to specific situations. In particular, the use of emotion became a positive predictor of perceived effort and number of training days.

Moreover, three studies investigated individual perceptions about COVID-19 with no-validated questionnaires. Clemente-Suarez et al. [23] found that elite athletes had high perception of social alarm, perceived a lack of support from institutions and had repercussions on training routines. Jagim et al. [38] highlighted a decrease in QoL due to lack of in-person support and social interaction with self-reported overall state of mental well-being approximately at a score of 50 on a scale 0–100. Pillay et al. [33] reported that 31% of investigated athletes felt unsure to return to sport because they were worried about the spread of virus.

## Discussion

This narrative review aimed to assess the effects of COVID-19 pandemic lockdown on the mental health of elite athletes, identifying common psychological repercussions and their correlates. The overall findings of this review showed that the lockdown imposed by COVID-19 pandemic impacted elite athletes’ mental health. Due to the unique situations, many authors reported increased level of stress, anxiety and psychological distress in general population, primary connected to long period of quarantine, lack of social contact and fear of contract the virus. Athletes’ population seems to have reported less impact of these factors than the general population probably thanks to the capacity to react to adverse events and the use of adequate coping strategies [25, 33]. The first model used in sports psychology was the Model of Sports Injuries [39, 40] that with the similarity in the interruption of activity and the inherent uncertainty of the return to normal sports conditions reproduces a condition comparable to COVID-19 stop for lockdown. This model added other outcomes such as self-efficacy and environmental factors to anxiety and stress factors [41]. Generally, the authors reported increased level of stress due to the uncertainty of COVID-19 spread, the disarray of calendar competitions with repercussion on physical fitness and training sessions. Even if, augmented levels of stress and anxiety were found by different authors, they did not highlight the presence of any pathological conditions. Leguizamo et al. [24] showed negative correlations between the use of coping strategies in athletes, mainly on cognitive restructuring and emotional calming, and the emotional states commonly identified as negative, such as depression, stress and anxiety. Clemente-Suarez et al. [23] confirmed low-to-no impact of confinement on anxiety levels of Olympic athletes thanks to the larger experience of high-performance athletes in coping with competition-related anxiety and the existence of higher cognitive resources. Contradictory, Szczypińska et al. [32] showed that not all the coping strategies used by the athletes had positive effects on their mental health, in fact they showed that behavioral disengagement and self-blaming had negative effect on the mood of athletes. Additionally, results were found by Ruffault et al. [25] where the measure of anxiety was contextualized to return to sport. The results showed that athletes with higher levels of anxiety also recorded higher scores of controlled motivations. This is in line with Self-Determination Theory returning to sport to avoid threats or to get external rewards is associated with anticipatory thoughts that lead to cognitive anxiety [42]. Authors noted that investigated athletes self-reported changed to their training frequency, time spent doing training activities, motivation to train, enjoyment from training, training effort compared to pre-COVID-19 activities. These changes were predominately associated with the cessation of in-person organized team practices, coach–athletes social interaction and impossibilities to use common training facilities as part of the COVID-19 lockdown measures. The self-reported decrease in perceived training intensity could reduce an athlete’s state of physical readiness when a return to sport is possible. The increased risk of future injuries is another major concern related to proper physical training for sport and compete again. A previous study reported that the lack of adequate preparatory strength and conditioning period led to detraining effects and predisposed athletes to a greater risk of injuries during explosive activities [43]. Nevertheless, athletes who followed training programs during lockdown period (either developed by their staff or by other sources) were less anxious, perceived more control, and were more intrinsically motivated to return to sport after the confinement period. This is in line with theoretical models of return to sport in the context of sport injury [44]. In fact, continuing to train, keeping in contact with the staff or other athletes, having daily goals and activities, are optimal conditions for being confident in the return to sport with a lower loss in performance and the pleasure of practicing sport and competing again [45].

As previously reported, the athletes’ profile, personality and ability to regulate emotions could play a fundamental role in the prevention of mental health impairments and to react to adverse events. Elite athletes showed a complex motivation profile, with both high controlled and autonomous regulations. This is in line with a recent analysis of motivational processes in Olympic medalists that highlighted elite athletes’ influence from external factors [46]. The restrained environment linked with the COVID-19 lockdown could have enhanced this perception of external control such as fear of the loss of financial support and contracts. Many authors [24, 31, 32], highlighted that mood sates profile such as Vigor was the highest, clearly indicating that athletes did not experience a decrease in their “energy perception” during lockdown and the ability to maintain a positive reframing help them to cope with COVID-19 situations. These traits matched with the so-called “Iceberg profile” always associated with high performance in athletes [47]. In fact, Clemente-Suarez et al. [23] stated that athletes presented a high perception of social alarm, but the concern about COVID-19 pandemic was medium, probably because of the high control perception and personal care to avoid contagion. Additionally, correlation analysis showed that neuroticism personalities perceived that confinement produced negative impact in the subjective performance with repercussion on training routines and perceived lack of institutional support. The negative emotions associated to this personality trait may cause a poor adaptive behavior during the confinement situation [48]. On the contrary, openness trait presented higher control and personnel care perceptions, more adaptive behavior to the confinement than the neuroticism ones. In this line, the psychological inflexibility showed to be related with poor adaptive responses to the confinement led to high negative perception of sport performance, training routines and feeling of more loneliness. Psychological inflexibility is a factor that has been related to worse states of health and less contextual adaptability [49]. For this reason, psychological training has proven to be relevant in elite athletes to maintain a positive mood and more flexible and adequate coping strategies that are fundamental parameters to achieve high sport performance [50–52].

Studies included in this review reported gender differences in the mental health of elite athletes highlighting the importance of using a gender-based psychological training. In fact, Fiorilli et al. [29] showed that female athletes had higher levels than males, designed to assess current subjective distress. These results are also confirmed by Parm et al. [30], which showed that females had higher distress in COVID-19 period than males. Additionally, Costa et al. [37], found that cognitive emotion regulation strategies showed specific gender differences as previously highlighted [53], with women reporting to use more “rumination” and “catastrophizing”. The reason of these behaviors could be found in the tendency of women to express their emotions more than men [54], and this period of social isolation might have been an obstacle to this expressivity. Also, response styles theory assumptions [55] could explain tendency for women to ruminate when experiencing negative mood or circumstances, whereas men tend to distract themselves. This rumination can, in turn, increase the possibility to remain in a negative mood and perceive the circumstances as impacting mind and body, with increased anxiety [56]. Women emerged also as being more able to put things into perspective, whereas men used more the cognitive strategy of planning (i.e., to think about what steps to take and how to handle the negative event). These differences are novel in literature, as the CERQ has not yet reached widespread use in the sporting field. Moreover, elite athletes scored higher values in “planning” and “acceptance”, and lower values in “self-blame”, is in line with Ashfar et al. [57]. Also, elite athletes have emerged as having better strategies to emotionally cope with stressful situations [58]. Indeed, Di Cagno et al. [36] reported that women showed higher avoidance levels than males. Contrariwise, male athletes used social contacts to resolve stressful situations such as the sports activity withdrawal. Shuer and Dietrich [59] found that avoidance could be a psychological defense to actively remove unpleasant thoughts and situations. Athletes are often familiar to the use of dissociative strategies to separate life problems from their performance. This approach, defined as “compartmentalization”, could have masked the presence of the psychological symptoms in female athletes [60].

We are conscious that this study had some limitations. First, we were able to analyze only sixteen papers of the initial ninety-nine, only those were eligible according to the criteria. However, the results are of possible interest for future lines of study and intervention in the psychological field. Second, this review considered only studies published in English language, such that relevant studies conducted in non-English samples have been omitted. Finally, we did not find relevant information about the Paralympic elite athletes. In fact, in this study, we analyzed papers focused on mental health and psychological distress in athletes during the lockdown period, starting from our results new research should study the best coping strategies for athletes to deal with stressors. COVID-19 consequences remain unclear and the pandemic continues to be a matter of concern for both the public and the scientific community, so our study could be a starting point to include athletes’ mental health evaluation after a COVID-19 diagnosis.

## Conclusion

Even if all the studies that we evaluated reported athletes higher perceived stress level, anxiety and psychological distress, the majority of participants were not substantially affected by the lockdown restrictions. The sports practice, in which athletes usually deal with stressful situations, such as competitive events, leads to achieve useful skills to manage anxiety and self-control in daily life. The athletes’ repeated exposure to exercise may have led to a stress response system adaptation and a negative cognitive appraisal. Elite athletes invest more in sport life and are able to better cope with stressful and uncertain situations [61]. Additionally, anxiety reduction techniques such as breathing exercises for physiological anxiety or mental exposure using imagery for cognitive anxiety may be taught to athletes with high anxiety [62]. Consequently, they may be able to transfer these skills from sport to the other life domains even during challenging times. Nevertheless, our review highlights the importance for coaches and physicians to keep under attention the level of stress and anxiety in elite athletes exacerbated from COVID-19 pandemic because these stressors can negatively influence the athletes’ performance and life. Sport psychologists and multidisciplinary interventions had to be implemented to early identify negative stressors and to help athletes to cope with these negative events to continue their careers and training in a safety way.
